# Effect of Conventional Humid–Dry Heating through the Maillard Reaction on Chemical Changes and Enhancement of In Vitro Bioactivities from Soy Protein Isolate Hydrolysate–Yeast Cell Extract Conjugates

**DOI:** 10.3390/foods13030380

**Published:** 2024-01-24

**Authors:** Rewat Phongphisutthinant, Pairote Wiriyacharee, Kongsak Boonyapranai, Sakaewan Ounjaijean, Sirinya Taya, Pornsiri Pitchakarn, Pattavara Pathomrungsiyounggul, Patamaphorn Utarat, Worachai Wongwatcharayothin, Chalermkwan Somjai, Supakit Chaipoot

**Affiliations:** 1 Multidisciplinary Research Institute, Chiang Mai University, Chiang Mai 50200, Thailand; rewat.p@cmu.ac.th (R.P.); sirinya.t@cmu.ac.th (S.T.); 2 Research Center of Microbial Diversity and Sustainable Utilization, Faculty of Science, Chiang Mai University, Chiang Mai 50200, Thailand; pairote.w@cmu.ac.th; 3 Faculty of Agro-Industry, Chiang Mai University, Chiang Mai 50100, Thailand; pattavara.p@cmu.ac.th (P.P.); sureepornutarat@gmail.com (P.U.); worachai.fst@gmail.com (W.W.); 4 Processing and Product Development Factory, The Royal Project Foundation, Chiang Mai 50100, Thailand; chalermkwansomjai@hotmail.com; 5 Research Institute for Health Sciences, Chiang Mai University, Chiang Mai 50200, Thailand; kongsak.b@cmu.ac.th (K.B.); sakaewan.o@cmu.ac.th (S.O.); 6 Faculty of Medicine, Chiang Mai University, Chiang Mai 50200, Thailand; pornsiri.p@cmu.ac.th

**Keywords:** Maillard reaction, soy, yeast, humid–dry heating, antioxidant activity, anti-inflammatory activity, angiotensin I-converting enzyme (ACE) inhibitory activity

## Abstract

This study investigated the formation of soy protein isolate hydrolysate–yeast cell extract (SPIH-YCE) conjugates through a humid–dry heating process and their impact on bioactivity. The incubation of SPIH-YCE samples at 60 °C and ~75% humidity for varying durations (0, 5, 10, 15, and 20 days) resulted in a significant decrease in reducing sugars and free amino acids, while the degree of glycation increased by approximately 65.72% after 10 days. SDS-PAGE analysis and size exclusion chromatography revealed the presence of peptides and glycoprotein molecules, with an increase in the distribution of larger peptide size chains. The conjugated SPIH-YCE (10 days) exhibited the highest antioxidant capacity compared to the other samples at different incubation times. A comparative study between SPIH-YCE (day 0) and SPIH-YCE after 10 days of incubation showed significantly higher anti-inflammatory and ACE inhibitory activities for the conjugates subjected to the humid–dry heating process. This suggests that SPIH-YCE conjugates could serve as an alternative substance with the potential to provide health benefits by mitigating or preventing non-communicable diseases (NCDs). This research highlights the importance of the Maillard reaction in enhancing bioactivity and offers insights into the alterations of the chemical structure of these conjugates.

## 1. Introduction

The Maillard reaction, also known as non-enzymatic glycosylation, is a complex chemical process involving amino acids and monosaccharides (reducing sugars) when exposed to heat. This reaction is responsible for browning and the development of complex flavors in a wide range of food products, including baked goods, dried fruits, roasted meats, and coffee. Glycosylation can result in the formation of conjugated or complex substances, such as protein–polysaccharide compounds, through covalent interactions. Both dry and wet heating conditions have been studied extensively in various research efforts [1,2,3,4,5,6,7]. An aging process involving dry heating at controlled temperatures and specific atmospheric humidity levels for several days to weeks is employed commercially in the production of black garlic. The manufacturing process involves maintaining temperatures within the range of 40–90 °C and humidity levels between 60 and 90% [8,9]. Furthermore, some studies on aged agricultural products, such as longan fruit and shiitake mushrooms, have demonstrated that the humid–dry heating process could enhance their bioactivities, particularly their antioxidative properties [10,11]. A mild heat–moisture process was also applied to raw paddy rice, and was able to impact in vitro starch digestibility [12]. A review by Sedaghat Doost et al. [2] reported novel techniques for the preparation of glycated products, which involve non-thermal alternative treatments such as ultrasonication, pulsed electric field, electrospinning, irradiation, and high pressure. Chemical synthesis techniques were also established to prepare polysaccharide–peptide/protein conjugations, including reductive amination, disulfide bond formation, the grafting-from method, the cyanogen bromide (CNBr) method, crosslinking, click chemistry, 4-(4,6-dimethoxy-1,3,5-triazin-2-yl)-4-methyl-morpholinium chloride (DMTMM) coupling, 1-ethyl-3-(3-dimethylaminopropyl) carbodiimide/N-hydroxysuccinimide (EDC/NHS) coupling, and the Ugi reaction [5]. In addition to these chemical synthesis methods, spontaneous chemical transformations of proteins and sugars during the Maillard reaction can lead to the production of protein–sugar graft polymer products, also known as glycated complex compounds. These products play a crucial role in enhancing both bioactive characteristics and functional properties. The bioactive properties of this complex encompass antioxidant activity, antibacterial activity, angiotensin I-converting enzyme (ACE) inhibitory activity, prebiotic effects, and acetylcholinesterase (AChE) inhibition activity. Simultaneously, the functional properties of the products, such as thermostability, delivery, solubility, encapsulation, emulsifying ability, foaming capacity, gelation, and thickening properties, are significantly improved [1,4,6,10,13,14].

Yeast stands as the most extensively employed microbe in various industrial processes, playing a particularly crucial role in the brewing, winemaking, distillation, and baking industries, and as a nutritional and flavoring ingredient in food products [15,16]. Non-viable yeast cells can be categorized as paraprobiotics, which have been demonstrated to regulate inflammation-inhibiting properties and promote immune-protective responses after consumption by both humans and animals [17]. Dried yeast cells are composed of various constituents, such as proteins, saccharides, amino acids, glucans, nucleotides, vitamin B complexes, and minerals [15,16]. Complex products of macro/micro molecules were obtained from yeast cell extraction using various production methods, including physical disruption, enzymatic degradation, autolysis, and plasmolysis. Additionally, yeast cell wall polysaccharides were extracted using enzymes, alkaline thermal treatment, microwaves, ultrasonication, high pressure, and cavitation under high pressure methods. Mannose oligosaccharides, glucan, chitin, and glycogen can be found in yeast (*Saccharomyces cerevisiae*) cell wall extracts, which can be utilized in food products, health products, biotechnology, cosmetics, and drug delivery, as well as in the feed industry and medical industry. Yeast extracts exhibit bioactive qualities such as having antioxidative, anti-aging, immune-boosting, prebiotic activity, anti-tumor, anti-inflammatory, antibiotic-substituent, and cholesterol-reducing properties [16,18,19,20,21,22]. Following the research by Marson et al. [23], which investigated hydrolyzed brewer’s yeast-derived Maillard conjugates in an aqueous condition combined with maltodextrin, the results found a substantial increase in the encapsulation efficiency of ascorbic acid and the enhancement of powder characteristics. Additionally, glucose glycated with yeast cell protein demonstrated the potential to serve as a carrier for curcumin, contributing to the antioxidant enhancement of these nanocomplexes [24].

Soybeans are legume seeds rich in protein that are readily available and affordable for consumers. Soy protein isolate stands as an essential product among soy-derived proteins, extracted from defatted soy meal and containing a high protein content of approximately 85−90% on a dry basis. The manufacturing process for this product involves solubilizing the protein under neutral or alkaline conditions. Subsequently, the solubilized protein is separated from the insoluble residue, followed by washing and neutralization, before being obtained as a dried powder [25,26,27]. Several studies on the glycation of soy protein with mono/polysaccharides have been conducted using both wet and dry techniques. These glycated products showed enhancements in the range of functional characteristics, such as solubility, thermal stability, emulsion and foaming qualities, structural flexibility, freeze–thaw stability, hydrophilicity, and increased antioxidant activity [7,27]. Yu et al. [28] illustrated that the conjugated compounds originating from soy peptide fractions (1–3 kDa) and D-xylose through the Maillard reaction displayed superior sensory attributes, including enhanced umami and meaty flavors. Nevertheless, achieving the optimal reaction conditions for soy protein–carbohydrate conjugation requires meticulous control of various parameters such as ratio, duration, temperature, relative humidity, and preparation techniques [27,29]. While various studies have examined soy protein isolates glycated with different saccharide molecules, no research has been conducted between crude yeast cell extract and soy protein hydrolysates. Consequently, this research purposed to investigate the effects of conventional humid–dry heating through the Maillard reaction on the chemical changes and the enhancement of in vitro bioactivities of these complex molecules. The findings could propose the potential for health benefits, with these natural compounds potentially serving as safe and sustainable functional and nutraceutical molecules in various health products aimed at preventing the development of non-communicable diseases (NCDs).

## 2. Materials and Methods

### 2.1. Materials and Chemicals

Instant dry yeast (Saf-Instant^®^ Blue Yeast, Lessaffre, Marcq-en-Barœul, France) and soy protein isolate (~87% dry basis of protein content from Food Great Products Co., Ltd., Bangkok, Thailand) were used as raw materials in this study. Alcalase enzyme from Bacillus licheniformis (>0.75 AU/mL) was purchased from Merck (Darmstadt, Germany). The chemical substances, reagents, and standards applied in this research included 1,4-dithiothreitol (Loba Chemie, Mumbai, India), 2,2-diphenyl-1-picrylhydrazyl (Sigma-Aldrich, Saint Louis, MI, USA), 2-mercaptoethanol (Merck, Malaysia), 3,5-minitrosalicylic acid (DNS) (Sigma-Aldrich, Karnataka Bengaluru, India), acetic acid (RCI Labscan, Bangkok, Thailand), boric acid (RCI Labscan, Bangkok, Thailand), bovine albumin fraction V (Sigma-Aldrich, Saint Louis, MI, USA), bromophenol blue (Sigma-Aldrich, Saint Louis, MI, USA), caprylic acid (octanoic acid) (Merck, Darmstadt, Germany), copper (II) sulfate pentahydrate (QReC, New Zealand), diclofenac (Visham Lifecare, India), dithiothreitol (LOBA Chemie, India), ethanol (RCI Labscan, Bangkok, Thailand), Folin–Ciocalteu reagent (Merck, Darmstadt, Germany), gallic acid (Sigma-Aldrich, Saint Louis, MI, USA), glycerol (RCI Labscan, Bangkok, Thailand), hexane (RCI Labscan, Bangkok, Thailand), hydrochloric acid (RCI Labscan, Bangkok, Thailand), L-lysine (Sigma-Aldrich, Saint Louis, MI, USA), methanol (AR grade) (RCI Labscan, Bangkok, Thailand), N-acetyl-L-cysteine (Merck, Darmstadt, Germany), O-phthalaldehyde (OPA) (Sigma-Aldrich, Tokyo, Japan), perchloric acid 70% (QRec, New Zealand), potassium sodium tartrate tetrahydrate (RCI Labscan, Bangkok, Thailand), potassium sulfate (RCI Labscan, Bangkok, Thailand), sodium acetate (RCI Labscan, Bangkok, Thailand), sodium carbonate (QRec, New Zealand), sodium citrate tribasic dihydrate (RCI Labscan, Bangkok, Thailand), sodium hydroxide (RCI Labscan, Bangkok, Thailand), sodium hypochlorite solution (4–6%) (Loba Chemie, India), sulfuric acid (RCI Labscan, Bangkok, Thailand), and trichloroacetic acid (TCA 99%) (Merck, Darmstadt, Germany).

A set of analytical amino acid standard mixtures, consisting of 17 amino acids, was obtained from Wako Pure Chemical Corporation (Osaka, Japan). Acetonitrile and methanol (HPLC-grade solvent) were obtained from RCI Labscan (Bangkok, Thailand). Sugar standards including D-allose, D-allulose, D-fructose, D-glucose, D-mannose, D-rhamnose, D-xylose, and sucrose were purchased from Sigma-Aldrich (Singapore). A protein standard mix of 15–600 kDa (Supelco, Darmstadt, Germany) was used to evaluate and monitor the performance of the size exclusion chromatography (SEC) column. All dilutions and solutions were prepared with demineralized water produced from a water purification system (Zeneer UP 900, Seoul, Republic of Korea).

### 2.2. Preparation of Soy Protein Isolate Hydrolysates

Soy protein isolate (SPI) suspension was prepared by mixing approximately 25 g of SPI powder in 1 L of deionized water, followed by gently stirring for 1 h at room temperature (~30 °C). Next, the suspension was adjusted to pH 8.0 (using 1.0 N NaOH), and alcalase was added at an enzyme-to-substrate ratio (E/S) of 1% (*v*/*w* protein) [30], and hydrolysis was operated at a temperature of 50 °C for 10 h using a mild agitation rate (15–20 rpm). The enzyme was inactivated at 95 °C for 15 min. The supernatant liquid was obtained by centrifugation at 8000 rpm and 25 °C for 10 min. Subsequently, the collected supernatant was placed on plastic trays, frozen at −20 °C for 24 h, and then transferred to a freeze-dryer (Harvest right, LCC, Salt Lake City, UT, USA). The freeze-drying process was conducted under temperature control ranging from around −35 °C to 25 °C, with the machine time function set for 20 h, resulting in the production of soy protein isolate hydrolysates (SPIH). The SPIH was stored in a freezer (−18 °C) for further analysis.

### 2.3. Crude Yeast Cell (Saccharomyces cerevisiae) Extract Preparation

Yeast peptone dextrose broth (YPDB) was used as a yeast medium. About 5 L was prepared by autoclaving at 121 °C for 15 min. Briefly, 250 g of instant dry yeast was inoculated in the yeast medium solution following the incubation time for 48 h at 25 °C under agitation at around 15–20 rpm. Yeast cell flocculation was harvested using a centrifuge (UNIVERSAL 320 R, Hettich, Massachusetts, Germany) at 7000 rpm/25 °C for 5 min. Yeast cells were desiccated at 60 °C using a hot-air oven (Memmert: Model UM 500, Schwabach, Germany) until the water content fell within the range of 1–2%, followed by pulverization to acquire yeast powder. Hexane was used to remove the oils from the yeast cell powder before extraction.

For the preparation of crude yeast cell extract (YCE), approximately 100 g of defatted yeast powder was used and subsequently extracted with 0.5 M HCl solution at a ratio of 1:10 (*w*/*v*). Then, the solution was processed in an autoclave (Hirayama: HICLAVE HVN-85, Saitama, Japan) at a temperature of 125 °C for 35 min. After cooling, the solution was neutralized to pH 7.0 using a 0.5 M NaOH solution and subsequently centrifuged at 8000 rpm for 10 min. The supernatant was collected and dried by using a freeze-dryer under the same conditions employed in the preparation of SPIH to attain the YCE for further analysis. The extract was kept at −18 °C to avoid chemical changes during storage.

### 2.4. Production of Soy Protein Hydrolysate–Yeast Cell Extract (SPIH-YCE) Conjugates through the Humid–Dry Heating Process

Approximately 10 g of SPIH and 10 g of YCE were dissolved and gently stirred in 100 mL deionized water. The SPIH-YCE suspension was lyophilized and then incubated at 60 °C in a glass desiccator previously equilibrated to ~75% relative humidity using a saturated NaCl solution [10]. This study was conducted at various incubation times of 0, 5, 10, 15, and 20 days, after which the treated samples were immediately packed in polyethylene bags and then stored at −18 °C in a freezer until analysis of their chemical and antioxidant characteristics. Subsequently, the selective SPIH-YCE conjugates, incubated under suitable conditions of incubation time, and a control sample (0 days), were chosen for the investigation of their bioactive activities (anti-inflammatory and ACE inhibitory activity) in comparison to the positive control drugs.

### 2.5. Measurement of Protein Content and Total Sugar and Reducing Sugar Content

The Dumas combustion assay 992.23 [31] was applied to measure the total nitrogen content of the extracts (SPIH and YCE). The total protein content in the extracts was determined using a conversion factor of 6.25 [32,33].

The total sugar content was quantified using the colorimetric phenol–sulfuric acid method described by DuBois et al. [34]. Crude extracts (SPIH and YCE) were diluted with distilled water. Approximately 1 mL of extract solution was mixed with 1 mL of 5% (*w*/*v*) phenol solution, followed by the addition of 5 mL of 96% sulfuric acid, one by one, to each tube with thorough agitation. After 10 min of incubation time, the tubes were placed in a water bath at 30 °C for 30 min. A blank sample was prepared with 1 mL of distilled water, and UV-vis spectroscopy (UV1800; Shimadzu, Japan) was performed to determine the absorbance at 490 nm. A standard curve using glucose (concentration range: 0.025–0.200 mg/mL) was established. 

A modified method for the analysis described by Gandhi et al. [35] was employed to prepare the 3,5-dinitrosalicylic acid (DNS) reagent. Standard glucose solutions (concentration range: 0.1–1.0 mg/mL) were used to establish a calibration curve. In a test tube, 1 mL of the crude extract was mixed with 4 mL of DNS reagent and covered with aluminum foil to prevent liquid loss while boiling in a water bath at approximately 90 °C for 5 min. The test tube was cooled rapidly in an ice bath followed by the addition of 10 mL of distilled water to stabilize the color. Absorbance measurements at 550 nm were taken for the samples using a UV–visible spectrophotometer with a blank sample consisting of distilled water to replace the crude extracts.

### 2.6. Sugar Analysis Using HPLC

Analysis of sugar standards (eight types) and all samples was carried out using the ShodexTM Capture the Essence method [10]. The HPLC technique was performed with a refractive index detector (RID) (Shimadzu, Kyoto, Japan), employing a HILICpak VG-50 4E Shodex HPLC column (4.6 mm I.D. × 250 mm length, Showa Denko, Tokyo, Japan). The mobile phase consisted of a mixture of acetonitrile, methanol, and water (in a ratio of 85:10:5, *v*/*v*). The test was maintained under isocratic conditions at a flow rate of 0.6 mL/min for 45 min by setting a column oven to 50 °C. Before injecting 10 µL aliquots for each run, the sample was diluted 2-fold with acetonitrile (in a 50:50, *v*/*v* ratio) and filtered through a membrane filter of 0.20 µm. The determination of the quantity for each sugar involved analyzing the retention time and linear plot of peak areas for different sugar concentrations.

### 2.7. Amino Acid Profiles and Peptide Molecular Weight Distribution 

#### 2.7.1. Analysis of Amino Acids using HPLC via Na-Type Determination

The determination of 17 amino acids was conducted using a post-column reaction method [6,10,36]. A column of Na-type sulfone group, Shim-pack AMINO-NA (100 mm length × 6.0 mm I.D.), with 5 μm particle size (P/N: 228–18837-91, Shimadzu, Japan), was utilized along with an RF-20A fluorescence detector (Shimadzu, Japan). The mobile phases, denoted as A, B, and C, were citrate buffers with pH values of 3.23 (A) and 10.0 (B), while C consisted of an aqueous solution of 0.2 M NaOH. Pre-column derivatizing reagents were prepared from N-Acetyl-L-cysteine and OPA. Running conditions included a flow rate of 0.4 mL/min and a column oven temperature of 60 °C. The sample was prepared by dilution with a sample diluent that was formulated by dissolving 9.8 g of tri-sodium citrate dihydrate in approximately 480 mL of DI water. Subsequently, 6.9 mL of 70% perchloric acid and 50 µL of octanoic acid were added. The final pH was set to 2.2, and the total volume was then adjusted to 500 mL. Prior to analysis, all samples underwent filtration through a 0.45 µm filter, and an injection volume of 10 µL was utilized. The quantification of amino acids was determined by calculating the retention time and linear trend of peak areas across various concentrations for each individual amino acid, which were then converted from micromole units to milligrams.

#### 2.7.2. SDS Polyacrylamide Gel Electrophoresis (Coomassie and Glycoproteins Stains)

Following the Laemmli protocol [37], SDS-PAGE was conducted using a 12% acrylamide separating gel at pH 8.8 and a 4% stacking gel at pH 6.8. To assess complexes or macromolecules, all samples were prepared with a 2-fold dilution in 2× Laemmli sample buffer, which included 26.3% (*w*/*v*) glycerol, 2.5% (*v*/*v*) 2-mercaptoethanol, 2.1% SDS, 0.01% bromophenol blue, and 65.8 mM Tris-HCl (pH 6.8). The samples (0.1% protein) were heated at 95 °C for 3 min, before loading 20 µL into the wells. After electrophoresis, the gel sheet was immersed in the mixed solution (40% methanol and 10% acetic acid) for 30 min. Subsequently, the gel was rinsed with deionized water and dyed using Coomassie brilliant blue solution (Bio-Rad, Hercules, CA, USA), after which the gel was subjected to destaining by soaking it overnight in a solution (25% methanol and 7% acetic acid) before scanning an image of the SDS-PAGE gel.

An alternative approach for identifying glycoproteins is using a detection kit (Sigma-Aldrich). A polyacrylamide gel was prepared following an identical process as for the prior peptide analysis, with variations in the handling of the samples. All samples were diluted in a sample buffer and then subjected to a boiling step for 3 min before being loaded onto the stacking gel. The gel was further processed following periodic acid-Schiff staining conditions, which involved fixing, washing, oxidation, staining, reduction, and washing, all carried out step by step. The magenta bands became visible after the staining in the final process. Bovine serum albumin (BSA) and peroxidase from horseradish (GP) were utilized as control components of complex peptides and glycoproteins, respectively. A standard reference molecular weight protein marker (2–250 kDa) (Bio-Rad, Hercules, CA, USA) was employed in this analysis.

#### 2.7.3. Analysis of Molecular Weight Distribution of Peptides Using Size Exclusion Chromatography

In the procedure described by Parrado et al. [38] and Zhang et al. [39], the molecular weight of soluble peptides was determined using the size exclusion chromatography (SEC) technique. An HPLC (Shimadzu, Kyoto, Japan) was performed with a UV-VIS detector at 280 nm and an SRT-C SEC-300 column (5 µm, 7.8 × 300 mm, Sepax Technologies, Inc., Newark, DE, USA). The column was equilibrated and eluted with 0.1 M sodium phosphate buffer (pH 7.0) in isocratic mode at a flow rate of 1.0 mL/min under temperature control at 30 °C. The samples were prepared with deionized water following filtration through a 0.45 µm membrane filter, in which the injection volume was 15 µL. A protein standard mixture was used to cover the range of 15–600 kDa, which consisted of 4 proteins: bovine thyroglobulin (~670 kDa), gamma globulins from bovine blood (150 kDa), albumin from chicken egg grade VI (44.3 kDa), and ribonuclease A type I-A from bovine pancreas (13.7 kDa), including a low-molecular-weight marker (*p*-aminobenzoic acid; *p*ABA). A calibration curve was established by plotting the logarithm of the molecular weight for these standards against their respective elution times. The estimation of the relative size distribution involved integrating the corresponding area under the chromatogram.

### 2.8. Degree of Glycation (DG)

The determination of the DG was conducted using the OPA assay [10], which estimated the depletion of functional amino groups. The OPA reagent, composed of 0.2 g OPA dissolved in 5 mL absolute ethanol, was then mixed with 125 mL of 0.1 M sodium tetraborate buffer (pH 9.75), 12.5 mL of 10% (*w*/*v*) sodium dodecyl sulfate (SDS), and 0.5 mL of 2-mercaptoethanol. The mixture was then diluted with distilled water to a final volume of 250 mL. About 3 mL of OPA reagent was added to the sample (50 μL) following agitation for 2 min at 25 °C. Absorbance was measured at 340 nm using a UV-vis spectrophotometer. A calibration curve was constructed using L-lysine (0.05–1 mM). The DG value was calculated according to Equation (1):

DG (%) = [(A_0_ − A*_t_*)/A_0_] × 100 (1)

where A_0_ represents the initial absorbance of the control sample, while A*_t_* signifies the absorbance observed after glycation for *t* days.

### 2.9. Investigation of In Vitro Bioactivities

#### 2.9.1. Analysis of Antioxidant Activity via ABTS, DPPH, and FRAP

All samples were evaluated for their antioxidant activity using three distinct techniques, including the ABTS radical cation assay, DPPH radical scavenging activity, and ferric reducing antioxidant power (FRAP), following the procedures outlined by Somjai et al. [6] and Chaipoot et al. [11].

For the ABTS radical cation assay, an oxidizing solution was prepared by blending 2.45 mM K_2_S_2_O_8_ with a 7 mM ABTS solution in 20 mM sodium acetate buffer (pH 4.5). The resulting mixture was left at room temperature in a dark place for 12–16 h to achieve a stable, dark blue-green radical solution. This oxidant solution was then diluted with 95% ethanol until it reached an absorbance of 0.70 ± 0.02 at 734 nm, serving as the working solution. Subsequently, 20 μL of each SPIH-YCE sample was introduced into 2 mL of the working solution, and the absorbance was determined at 734 nm after incubating the solution at ambient temperature, kept in darkness for a duration of 6 min. The ABTS radical inhibition capacity was assessed using a gallic acid calibration curve, and the findings are presented in milligrams of gallic acid equivalents (GAEs) per 100 g of the sample.

In the technique for DPPH radical scavenging activity, 1 mL of the SPIH-YCE sample was mixed with 2 mL of 0.2 mM DPPH free radical (2,2-diphenyl-1-picrylhydrazyl) dissolved in 80% methanol solution. The mixture was thoroughly stirred and left at room temperature without light for 30 min. Subsequently, the absorbance at 517 nm was measured using a UV–visible spectrophotometer (UV1800; Shimadzu, Japan). A blank was prepared following the same method, substituting distilled water for the sample. A calibration curve was generated using Trolox, and the antioxidant activity was quantified in milligrams of Trolox equivalents (TEs) per 100 g of the sample.

For the measurement of ferric reducing antioxidant power, FRAP reagent solution was prepared by combining 2.5 mL of 10 mM TPTZ solution in 40 mM HCl, 2.5 mL of a 20 mM FeCl_3_·6H_2_O solution, and 20 mL of a 300 mM acetate buffer (pH 3.6). This mixture was then kept at 37 °C for a duration of 30 min. Afterwards, 50 μL of SPIH-YCE sample was introduced into 750 μL of the FRAP solution and left in the darkness for 30 min. The alteration in the color was assessed at a wavelength of 593 nm. A standard curve was established using FeSO_4_·7H_2_O, and the outcomes are expressed in milligrams of FeSO_4_ equivalent per 100 g of the sample. 

#### 2.9.2. Analysis of Anti-Inflammatory Activity Using the Protein Denaturation Method

The ability of the selected SPIH-YCE conjugates and SPIH-YCE (day 0) to restrain protein denaturation was analyzed following the procedure outlined in Williams et al. [40] with minor adjustments. Three different concentrations (1.0, 10.0, 100.0 mg/mL) were prepared for each sample in phosphate buffer (0.05 M, pH 6.3). Approximately 500 µL of each sample was mixed with 2.0 mL of 10 mg/mL bovine albumin fraction V. The reaction mixture underwent initial incubation at 37 °C for 15 min, followed by subsequent heating at 70 °C for 5 min. After cooling to room temperature, the A660 of the mixture was measured. Diclofenac, a drug, served as the positive standard. All tests were prepared in triplicate. The percentage of inhibition of albumin denaturation was calculated according to the following Equation (2):

Inhibition (%) = [(A_0_ − A*_S_*)/A_0_] × 100 (2)

where A_0_ is the absorbance of the control, and A*_S_* is the absorbance of the sample or the positive control.

#### 2.9.3. Angiotensin I-Converting Enzyme (ACE) Inhibitory Activity

The selected conjugated SPIH-YCE and SPIH-YCE (day 0) were tested for ACE inhibitory activities at different concentrations (0.1, 1.0, 10.0 mg/mL) using an ACE kit-WST (Dojindo Laboratories, Kumamoto, Japan), as described in Chaipoot et al. [41]. The assay was carried out following the manufacturer’s instructions. Absorbance readings at 450 nm were quantified using a microplate reader. Equation (3) was utilized to calculate the ACE inhibitory activities of the samples as follows:

ACE inhibitory activity (%) = [(A_blank1_ − A_sample_)/ (A_blank1_ − A_blank2_)] × 100 (3)

where A_blank1_ is the absorbance of the positive control (without ACE inhibition), A_sample_ is the absorbance of the sample, and A_blank2_ is the absorbance of the reagent blank.

### 2.10. Statistical Analysis

Statistical evaluations were carried out utilizing SPSS software version 17.0 (SPSS Inc., Chicago, IL, USA). To assess significant distinctions, a one-way analysis of variance (ANOVA) was conducted. Subsequently, Tukey’s multiple comparisons test was employed. A significance threshold of *p* ≤ 0.05 was applied.

## 3. Results and Discussion

### 3.1. Chemical Characteristics of Soy Protein Isolate Hydrolysates (SPIH) and Yeast Cell Extract (YCE)

The total sugar and reducing sugar contents in both SPIH and YCE were subjected to analysis. The SPIH contained 2.46 g/100 g of total sugar and 0.02 g/100 g of reducing sugar, while YCE exhibited higher concentrations of both total and reducing sugars, with values of 17.41 g/100 g and 12.67 g/100 g, respectively. Other mono/disaccharides, including mannose, glucose, fructose, xylose, rhamnose, allulose, and allose, were also examined. It was observed that SPIH did not contain any of these sugars, whereas YCE exhibited mannose, glucose, and rhamnose at levels of 7.01, 6.16, and 0.44 g/100 g, respectively (Table 1). Comparable findings from a study by Krisdaphong et al. [42] revealed that yeast extract obtained through the autolysis process exhibited approximately 99% glucose along with small amounts of mannose and rhamnose. Despite using the same extraction method and raw materials, a substantial variation in the composition of the acquired yeast extract might exist, which could pose challenges in controlling the final products. The yeast lysis conditions, including pH (5.0–6.5) and temperature (50–70 °C), were carefully controlled to obtain the desired extract [16,22]. Regarding soy protein hydrolysates, they also contain a carbohydrate portion, with sucrose and stachyose as the primary components, along with minor proportions of raffinose, fructose, glucose, etc., depending on batch production [43]. 

**Figure 1:**
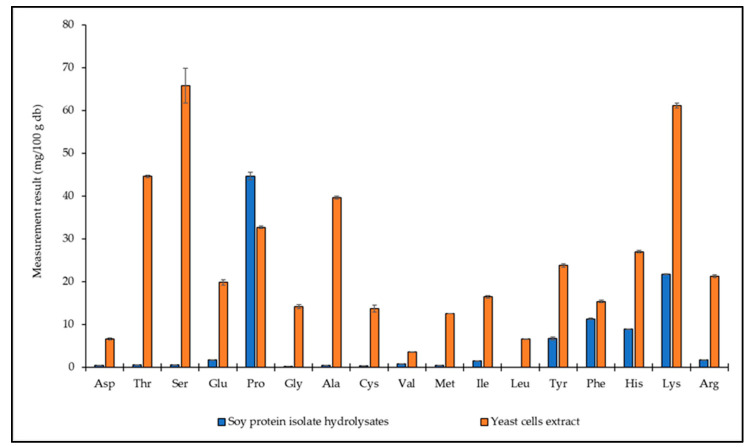
Amino acid composition in extract solutions of soy protein isolate hydrolysates (SPIH) and yeast cell extract (YCE): Asp (aspartic acid), Thr (threonine), Ser (serine), Glu (glutamic acid), Pro (proline), Gly (glycine), Ala (alanine), Cys (cysteine), Val (valine), Met (methionine), Ile (isoleucine), Leu (leucine), Tyr (tyrosine), Phe (phenylalanine), His (histidine), Lys (lysine), Arg (arginine).

The total protein content of SPIH and YCE showed both extracts containing protein quantities of 58.31 g/100 g and 79.57 g/100 g, respectively. Additionally, both extracts were investigated concerning 17 amino acids using the HPLC technique. SPIH was found to comprise five predominant amino acids, including Pro, Lys, Phe, His, and Tyr at concentrations of 44.62, 21.74, 11.27, 8.89, and 6.70 mg/100 g, respectively. The remaining amino acids were detected within a range of 0.15–1.71 mg/100 g. Notably, Leu was not detected in the extract. Focusing on the YCE, it contained all types of amino acids with the highest content of Ser (65.77 mg/100 g) and Lys (61.12 mg/100 g). Other amino acids included Thr, Ala, Pro, His, Tyr, Arg, Glu, Ile, Phe, Gly, Cys, Met, Asp, Leu, and Val, which were found in the extract with average values of 44.57, 39.63, 32.65, 26.97, 23.75, 21.28, 19.79, 16.43, 15.33, 14.12, 13.67, 12.46, 6.60, 6.54, and 3.53 mg/100 g, respectively (Figure 1). According to Djemal et al. [37], Arg, Leu, Lys, Glu, and Asp were the types of amino acids that were abundant in soy hydrolysates, with the molecular weight distribution of peptides being below 0.5 kDa. Commercial soy protein hydrolysates were composed of peptides/amino acids, carbohydrates, vitamins, lipids, and minerals. The protein content exhibited variability within a range of 56–58%, influenced by batch-to-batch differences and soybean cultivation [43,44]. Yeast extract primarily contained umami-flavor amino acids such as Glu, Ala, Gly, and Asp, along with various other types of amino acids like Ser, Val, and Pro depending on the variety of yeasts. The free amino acid content in yeast extract comprised approximately 35–40% of the total protein, with 40–45% of yeast oligopeptides having a molecular weight ranging from 2 to 3 kDa [16,22,45,46]. Based on the chemical composition of both SPIH and YCE, the sugar–protein conjugates formed through the Maillard reaction may interact synergistically, with the reaction rate being limited by the quantity of the sugar.

### 3.2. Chemical Changes in the SPIH-YCE Conjugation through the Humid–Dry Heating Process

#### 3.2.1. Changes in the Sugar Component in the SPIH-YCE Samples after the Humid–Dry Heating Procedure

**Figure 2:**
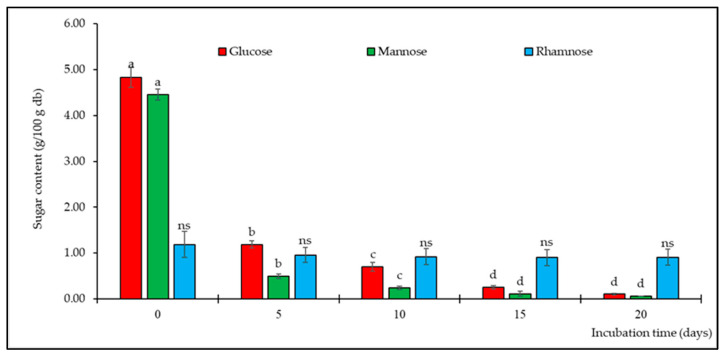
Amount of glucose, mannose, and rhamnose in the SPIH-YCE samples during the humid–dry heating procedure at different incubation times. Different lowercase letters indicate significant differences among the different incubation times in amount of glucose, mannose, and rhamnose (*p* ≤ 0.05); ns indicates not significant (*p* > 0.05).

Data on three types of monosaccharides, analyzed using a Shodex HPLC column, are shown in Figure 2. The unincubated SPIH-YCE (day 0) was used as the control sample, which was composed of glucose, mannose, and rhamnose in amounts of 4.83, 4.46, and 1.19 g/100 g, respectively. It was observed that there was a significant reduction in glucose and mannose content during the first 15 days of incubation (*p* ≤ 0.05). On day 5 of incubation, the amount of glucose and mannose in the sample decreased significantly by approximately 75% and 88%, respectively. However, the quantities of these sugars remained unchanged (*p* > 0.05) when incubated for more than 15 days. The values for glucose ranged from 0.10 to 4.83 g/100 g and those for mannose ranged from 0.06 to 4.46 g/100 g. In contrast, the rhamnose content remained consistently stable throughout the entire incubation period, ranging from 0.91 to 1.19 g/100 g.

Reducing sugars could dwindle during the heat treatment, leading to the formation of sugar–amino acid crosslinks. The glycation through the Maillard reaction could be influenced by several factors, including temperature, time, pH, types of sugars, and proteins. Among the common hexoses, mannose demonstrated an elevated glycosylation reaction rate with α-lactalbumin, in contrast to glucose, possibly due to variations in the balance between the open and closed ring configurations of saccharide structures [47]. Various reducing sugars exhibited different reactivity levels, with pentose sugars showing higher reactivity compared to hexose sugars. Additionally, larger saccharide molecules led to a decrease in the formation of amino–sugar conjugates through covalent bonds with increased heating time [27,48,49]. However, the content of rhamnose in this study remained unchanged during incubation time, whereas research by Cardoso et al. [47] reported a high rate of glycation reactions.

#### 3.2.2. Changes in Amino Acid and Peptide Molecular Weight Content in the SPIH-YCE Samples after the Humid–Dry Heating Procedure

The amino acid components in the SPIH-YCE samples during the humid–dry heating procedure are displayed in Table 2. All samples were found to contain 17 amino acids, with the control sample exhibiting the highest quantity compared to the treated SPIH-YCE samples. The total amino acid content, which represents the sum of 17 different amino acids, was 261.84 mg/100 g. Among these amino acids, those found in descending order in the SPIH-YCE (0 day) were Lys, Pro, Ser, Thr, Ala, Arg, His, Tyr, Glu, Phe, Ile, Met, Cys, Gly, Leu, Asp, and Val. The amino acid profiles in the SPIH-YCE samples underwent a significant decrement after incubation for 5 days (*p* ≤ 0.05), with each type of amino acid being discernably reduced. However, those amino acids exhibited a slight decline and remained stable after 10 days of continued incubation. Approximately 10.11% of amino acids in the SPIH-YCE sample remained after 5 days of the humid–dry heating procedure, while the remaining amino acids ranged from 5.86% to 5.05% during the 10 to 20-day processing period. 

The Maillard reaction occurred through the humid–dry heating procedure, leading to a decrease in the quantity of free or protein-bound amino acids. This diminution was a result of the interaction between the terminal amino group and the carbonyl group of reducing sugars, resulting in the formation of conjugated compounds or Maillard reaction products. In particular, the intermediate stage of the Maillard reaction offers several pathways including dehydration, oxidation, enolization, acid hydrolysis, fragmentation, and free radical reactions, potentially leading to a wide range of complex and valuable compounds [5]. Furthermore, it might be a consequence of the non-covalent interactions between amino acids and phenolic components, leading to the formation of phenol–protein conjugates. These conjugates can form various types of crosslinks, including hydrophobic, electrostatic, hydrogen, and Van der Waals force interactions [6,7,10,27,50].

**Figure 3:**
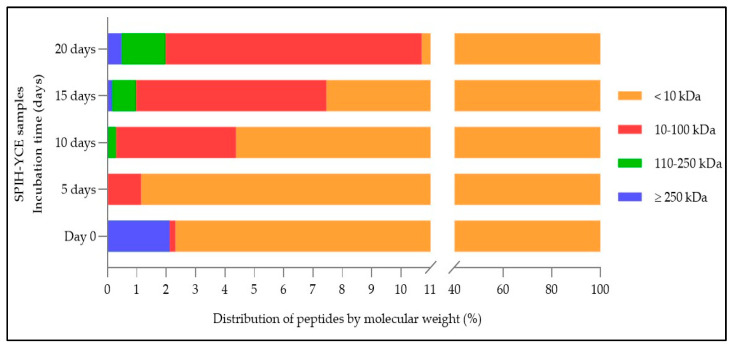
Distribution of peptide molecular weight, analyzed using HPLC, in SPIH-YCE samples during the humid–dry heating procedure at different incubation times.

All SPIH-YCE samples were evaluated for the distribution of peptide molecular weight using HPLC, as seen in Figure 3. Molecular weight peptides were separated into four size categories (>250 kDa, 101–250 kDa, 10–100 kDa, and <10 kDa). The SPIH-YCE sample without incubation contained approximately 97.68% of peptides with a size of <10 kDa, followed by 2.12% of peptides >250 kDa, and 0.20% of peptides in the 10–100 kDa size range. Peptide chains larger than 250 kDa decreased after the 5th day of incubation, and these larger peptides were distributed slightly more in the SPIH-YCE conjugates after 10 days of incubation, with a size distribution ranging from 0.02% to 0.48%. Conversely, their distribution increased after 5 days of incubation when focusing on peptides with a size of less than 10 kDa, but decreased as the remaining incubation duration continued, with the molecular weight distribution declining from 98.85% to 89.30%. In addition, only peptides with a molecular weight between 10 and 100 kDa increased in percentage distribution from 0.20% to 8.73% after 5 days of processing. Peptides ranging from 100 to 250 kDa occurred in the SPIH-YCE conjugates on day 10 of the process, and they exhibited a slight increase during the incubation period.

**Figure 4:**
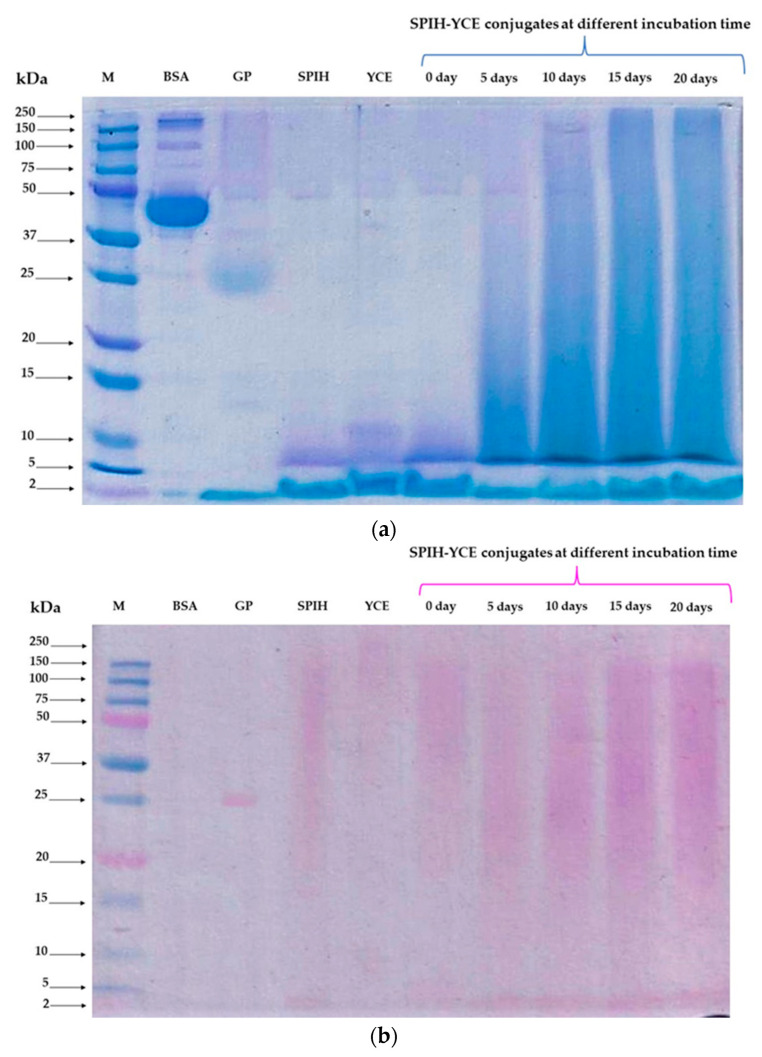
SDS-PAGE gel image of SPIH-YCE samples during the humid–dry heating procedure at different incubation times; (**a**) Coomassie brilliant blue; (**b**) glycoprotein stain. Lane M: protein marker; lane BSA: bovine serum albumin; lane: GP: glycoprotein (horseradish peroxidase); SPIH: soy protein hydrolysates; YCE: yeast cell extract; 0 days–20 days: SPIH-YCE conjugates at different incubation times.

The complexity and macro size of the peptide molecular aggregates were evaluated using the SDS-PAGE technique. Gel images for all SPIH-YCE samples, bovine serum albumin (BSA), and glycoproteins (GP) are displayed in Figure 4. Figure 4a shows a gel stained with Coomassie brilliant blue for peptide separation and visualization, while Figure 4b exhibits a gel stained with fuchsin-sulfite for glycoproteins or sugar–protein conjugation molecules. The samples of SPIH, YCE, and the SPIH-YCE (day 0) showed similar SDS-PAGE band patterns, suggesting peptides with similar molecular weights or within the same size range. Their peptide bands were predominantly below 15 kDa, with a small fragment appearing about 50 kDa. After the humid–dry heating process, the treated samples of SPIH-YCE exhibited an increase in smear blue bands in the middle to the top zone of the acrylamide gel as the incubation time extended, potentially indicating the formation of high-molecular-weight peptides or conjugated compounds. Another glycoprotein gel analysis revealed the presence of magenta smear bands in both samples, with and without the humid–dry heating operation. It was observed that the intense magenta smear zones were primarily present within the molecular weight range of 15–150 kDa, with some bands smaller than 5 kDa. When comparing the control SPIH-YCE sample (day 0) to all the treated samples, the magenta smear zones faded slightly during 5 days and 10 days of incubation. On the other hand, the magenta smear bands of glycoprotein became slightly more intense after 15 days of the procedure, possibly implying the establishment of sugar–peptide compounds. 

According to Conti et al. [51] and Djemal et al. [43], soy protein hydrolysates exhibited peptides with molecular masses ranging from 0.5 to 10 kDa, while yeast oligopeptides were characterized by a molecular weight within the range of 2–3 kDa [16]. The increase in peptide molecular weight through humid–dry heating incubation could be attributed to glycosylation between peptides and saccharides, resulting in the formation of conjugated compounds, as evidenced by the presence of polydisperse bands in the middle to the top of the SDS-PAGE gel, along with a broader distribution of larger peptide chain masses [6,52]. An increase in glycoprotein content was observed as the incubation time increased due to the Maillard reaction. Nevertheless, it is important to highlight that glycoprotein components were inherently present in both SPIH and YCE, as shown in a gel stained with fuchsin-sulfite. Silva Araújo et al. [53] characterized the β-glucan and mannoprotein from spent brewer’s yeast cells, with the mannoprotein possessing peptide molecular weights of 58 kDa and 64 kDa. Approximately 90% of the sugars in mannoprotein structures consisted predominantly of mannose types, which were found primarily in the outermost layer of yeast cells [54]. Furthermore, a study by Li et al. [55] discovered that soybean protein isolates contained glycoproteins with the various structural features of N-glycoforms, including oligomannose-type glycans.

#### 3.2.3. Degree of Glycation (DG) in SPIH-YCE Samples after the Humid–Dry Heating Procedure

**Figure 5:**
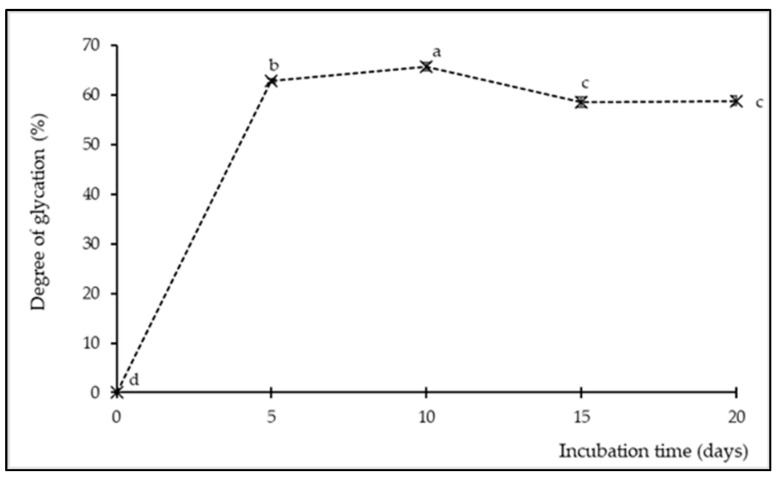
Degree of glycation of SPIH-YCE samples during the humid–dry heating procedure at different incubation times. Different lowercase letters indicate significant differences among the different incubation times in degree of glycation (*p* ≤ 0.05).

The DG was analyzed using the spectrophotometric OPA assay, and the results are displayed in Figure 5. The SPIH-YCE sample (day 0) served as the control (A_0_) and was designated with a DG value of zero, representing the initial quantity of free amino groups in the sample. The decrease in these amino groups during the incubation time defined an increase in the DG associated with the glycosylation grafting reaction. The DG increased significantly with incubation time, reaching a maximum value of approximately 65.72% after 10 days of incubation (*p* ≤ 0.05). Subsequently, the values decreased slightly and remained relatively constant, ranging from 58.55% to 58.86% after 15 days of incubation.

Grafting reactions between the side chains of amino acids and the carbonyl sites of saccharides and/or reducing sugars in the SPIH-YCE samples were carried out using the humid–dry heating procedure. These results were in line with the observed reduction in the levels of both amino acids and monosaccharides in the previous analysis. However, the slight decrease in the DG may have contributed to the instability in the grafting polymerization. The unchanged DG could be attributed to the limited quantity of sugar in the sample. The results resemble those of Somjai et al. [10], who studied aged longan pulp and observed an increase in the DG during incubation through the moist–dry heating process. Many studies have shown that glycated protein compounds can positively impact bioactivities and functional properties [1,7,14].

### 3.3. Effect of the Humid–Dry Heating Process on In Vitro Bioactivities of SPIH-YCE Conjugates

#### 3.3.1. Effect on Antioxidant Activities

Three methods were used to test the antioxidant activity of all samples, and the results are shown in Table 3. The SPIH-YCE samples demonstrated an increase in activities as the incubation time increased (*p* ≤ 0.05), particularly notable at 5 and 10 days of incubation for ABTS. In the cases of DPPH and FRAP, the SPIH-YCE conjugates exhibited significantly elevated activities at 10 days of incubation. The values obtained were 750.06 mg GAEs/100 g for ABTS, 904.58 mg TEs/100 g for DPPH, and 5102.95 FeSO_4_/100 g for FRAP. However, it was observed that the antioxidative capacities of conjugated SPIH-YCE samples declined significantly when incubated for longer than 10 days.

Several studies have explored Maillard reaction products and conjugated compounds formed during the heating process in the presence of moisture. These complex compounds played a role in imparting antioxidative properties, involving interactions with radical chains through hydroxyl and pyrrole groups. Therefore, the increase in antioxidant activity in SPIH-YCE conjugates might be attributed to their ability to degrade hydrogen peroxide, trap reactive oxygen species, exhibit metal-chelating capacity, and facilitate electron transfer [10,11,56]. Yeast extract and soy protein hydrolysates exhibited spontaneous antioxidant capacity. The presence of polysaccharide components in yeast cell walls could reveal their capability to neutralize hydroxyl free radicals and superoxide anions [22]. On the other hand, certain peptides in soy protein hydrolysates have displayed antioxidant among other bioactivities, for instance anti-cancer, immunomodulatory, and anti-hypertensive [51]. In addition, peptides could improve their bioactivity, particularly antioxidant activity, when they interacted with saccharides during the Maillard reaction [1,6,7,13,14,57]. Nevertheless, an excessively long incubation period may lead to a decrease in antioxidative ability, consistent with the findings of previous studies on reduced glycation levels. Another reason for the weakened antioxidative ability may be attributed to the partial decomposition, conformational changes, or thermal instability of Maillard reaction products when exposed to excessive heat [58,59].

#### 3.3.2. Effect of SPIH-YCE Conjugates on Anti-Inflammatory and ACE Inhibitory Activity 

**Figure 6:**
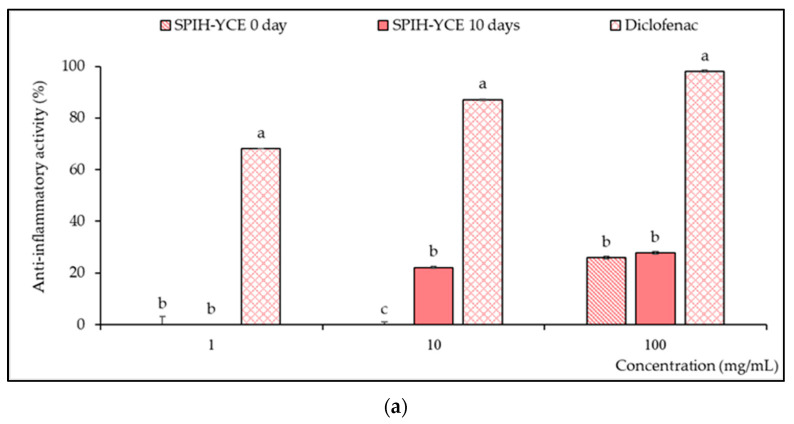

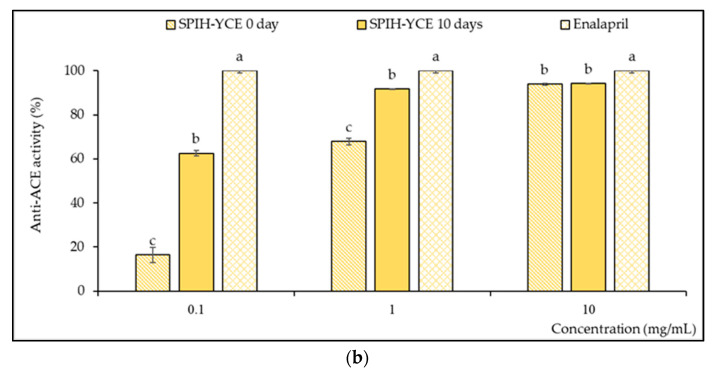
SPIH-YCE conjugates affected (**a**) anti-inflammatory activity and (**b**) ACE inhibitory activity compared to the control sample (SPIH-YCE on day 0) and positive controls (diclofenac; enalapril). Different lowercase letters indicate significant differences among the SPIH-YCE conjugates, control sample, and positive control (*p* ≤ 0.05).

The samples of SPIH-YCE (control) and SPIH-YCE conjugates were investigated for protein anti-denaturation activity at 10 days using a method that assessed anti-inflammatory effects. This method utilized bovine serum albumin (BSA) as an antigen to simulate type III hypersensitivity reactions, and evaluated the ability of the extract to prevent protein denaturation, which is associated with tissue injury and a reduction in inflammation [40,60]. This study demonstrated that SPIH-YCE conjugates exhibited the potential to inhibit BSA denaturation at 10 days, with concentrations exceeding 10 mg/mL resulting in inhibition rates ranging from 22.10% to 27.76%. In contrast, the SPIH-YCE sample without incubation demonstrated anti-inflammatory activity at concentrations exceeding 100 mg/mL, resulting in a 25.91% inhibition rate. When calculated as the IC50 value, both exhibited values exceeding 100 mg/mL to achieve a 50% inhibition rate, whereas the IC50 value for the diclofenac was 0.66 mg/mL. At a concentration of 10 mg/mL, the inhibition rate of SPIH-YCE conjugates (10 days) subjected to the humid–dry heating procedure was significantly higher than that of the sample without the process (*p* ≤ 0.05), as seen in Figure 6a. 

ACE inhibition was also analyzed for these two samples (Figure 6b). ACE is an enzyme that catalyzes the conversion of angiotensin I to generate angiotensin II, which exhibits strong vasoconstrictor activity. It also cleaves the vasodilator bradykinin, contributing to an increase in blood pressure. Thus, ACE inhibition was used as a preliminary step before in vivo confirmation to assess its potential to prevent hypertension [41]. The results showed that an increase in the concentration of the samples from 0.1 to 1.0 mg/mL led to a significant increase in anti-ACE activity (*p* ≤ 0.05). After 10 days of incubation, the conjugated SPIH-YCE exhibited a higher inhibition rate than the unincubated SPIH-YCE sample. The percentage of ACE inhibition for SPIH-YCE conjugates ranged from 62.54% to 94.16%, while the control sample showed inhibition in a range from 16.39% to 93.93%. The IC50 values for SPIH-YCE (without incubation) and SPIH-YCE conjugates were 0.68 mg/mL and <0.10 mg/mL, respectively. Enalapril was used as a positive control with an IC50 value of 0.03 µg/mL. In addition, there was no significant difference in the ACE inhibition rate when using samples at a concentration of 10 mg/mL.

Kitts et al. [57] demonstrated that sugar–amino acid products generated via the Maillard reaction exhibited the potential to restrain oxidative stress and inflammation in interferon gamma-stimulated interleukin-8 and phorbol ester (PMA)-induced Caco-2 cells. Furthermore, whey protein isolates glycated with galactose exhibited a higher concentration of organic acids compared to non-glycated whey protein and galactose during fermentation, which indicated an improvement in anti-inflammatory properties. This observation might be attributed to the presence of acetic acid and formic acid degradation products of the Maillard reaction [61]. Li et al. [62] investigated the glycation of salmon myofibrillar protein with reducing sugars through the Maillard reaction and found that it enhanced the anti-inflammatory activity. 

The Maillard reaction or glycosylation between peptides and sugars could also enhance ACE inhibitory activity, possibly due to the formation of carbonyl–ammonia condensation in complex Maillard reaction products. This improvement might result from the chelation of zinc metal transition or the reducing activity of peptides modified through the Maillard reaction by the heating process [63,64,65]. However, prolonged reaction times could diminish this activity [1,14,66]. Nonetheless, bioactive compounds derived from soybeans have naturally demonstrated potent in vitro ACE inhibitory activity and effectiveness against inflammatory mediators [67,68]. Additionally, the β-glucan and mannan polysaccharides found in the cell wall of yeast revealed health-promoting properties related to scavenging free radicals, delaying aging, and reducing lipid levels and blood cholesterol, as well as anti-inflammatory effects [17,19,22].

## 4. Conclusions

This study investigated the effects of conventional humid–dry heating under the Maillard reaction on the chemical changes and the enhancement of in vitro bioactivities of complex compounds using a mixture of soy protein hydrolysates and yeast extract as the primary natural ingredients. The results indicated that incubating the SPIH-YCE sample for 10 days at 60 °C under controlled relative humidity at ~75% created suitable conditions for producing conjugated SPIH-YCE compounds. This was associated with a reduction in reducing sugars (glucose and mannose) and amino acids, as well as an increase in the degree of glycation. SDS-PAGE gel analysis of SPIH-YCE after the incubation process revealed the presence of peptides and glycoprotein molecules, leading to an increment in the distribution of larger-sized molecular peptides. Furthermore, these SPIH-YCE conjugates exhibited the highest antioxidant capacity when compared to the other samples at different incubation times. A comparative study between SPIH-YCE (day 0) and SPIH-YCE after 10 days of incubation time was conducted to evaluate their anti-inflammatory and ACE inhibitory activity. It was observed that the inhibition rate in both tests for SPIH-YCE conjugates (10 days) was significantly higher than that of the sample without the humid–dry heating process. Therefore, all the findings concerning SPIH-YCE conjugates suggest the potential for health benefits in aiding the mitigation or prevention of NCDs, particularly hypertension and diseases related to inflammation. They also serve as natural and sustainable functional and nutraceutical components. Nevertheless, comprehensive research on this conjugated compound should be continued, encompassing the chemical structure, sequences of conjugated components, and cytotoxicity assessments, and additional confirmatory studies related to the conjugated system should be conducted.

## Figures and Tables

**Table 1:** Sugar content in the extract solutions of soy protein isolate hydrolysates (SPIH) and yeast cell extract (YCE).

Sugar Content (g/100 g db)	Soy Protein Isolate Hydrolysates (SPIH)	Yeast Cell Extract (YCE)
Total sugar	2.46 ± 0.13	17.41 ± 0.25
Reducing sugar	0.02 ± 0.01	12.67 ± 0.13
Sucrose	ND	ND
Mannose	ND	7.01 ± 0.01
Glucose	ND	6.16 ± 0.47
Fructose	ND	ND
Xylose	ND	ND
Rhamnose	ND	0.44 ± 0.04
Allulose	ND	ND
Allose	ND	ND

Data represented as mean ±SD of three replicates; ND = not detected; db = dry basis.

**Table 2:** Composition of amino acids in the SPIH-YCE samples during the humid–dry heating procedure at different incubation times.

Amino Acids (mg/100 g db)	Incubation Time (Days)				
	0	5	10	15	20
Asp	3.96 ± 0.16 ^a^	3.04 ± 0.16 ^b^	2.51 ± 0.15 ^c^	2.42 ± 0.26 ^c^	2.43 ± 0.12 ^c^
Thr	21.63 ± 0.90 ^a^	1.18 ± 0.36 ^b^	0.31 ± 0.02 ^c^	0.27 ± 0.01 ^c^	0.26 ± 0.02 ^c^
Ser	33.89 ± 0.88 ^a^	0.40 ± 0.02 ^b^	0.35 ± 0.02 ^b^	0.28 ± 0.02 ^b^	0.27 ± 0.01 ^b^
Glu	12.39 ± 1.18 ^a^	2.45 ± 0.11 ^b^	1.42 ± 0.05 ^c^	0.93 ± 0.08 ^c^	0.89 ± 0.08 ^c^
Pro	36.31 ± 0.87 ^a^	0.26 ± 0.03 ^b^	0.28 ± 0.02 ^b^	0.26 ± 0.02 ^b^	0.27 ± 0.01 ^b^
Gly	5.54 ± 0.38 ^a^	0.54 ± 0.11 ^b^	0.30 ± 0.01 ^b^	0.26 ± 0.02 ^b^	0.25 ± 0.02 ^b^
Ala	19.74 ± 0.46 ^a^	1.76 ± 0.23 ^b^	1.19 ± 0.02 ^c^	1.05 ± 0.05 ^c^	1.01 ± 0.12 ^c^
Cys	7.10 ± 0.11 ^a^	1.21 ± 0.19 ^b^	0.92 ± 0.01 ^b^	0.84 ± 0.07 ^b^	0.81 ± 0.06 ^b^
Val	1.51 ± 0.17 ^a^	0.08 ± 0.03 ^b^	0.07 ± 0.01 ^b^	0.06 ± 0.02 ^b^	0.07 ± 0.02 ^b^
Met	7.20 ± 0.42 ^a^	0.65 ± 0.10 ^b^	0.63 ± 0.06 ^b^	0.46 ± 0.03 ^c^	0.46 ± 0.02 ^c^
Ile	7.84 ± 0.18 ^a^	0.64 ± 0.09 ^b^	0.66 ± 0.03 ^b^	0.52 ± 0.03 ^b^	0.50 ± 0.05 ^b^
Leu	4.03 ± 0.17 ^a^	0.54 ± 0.06 ^b^	0.40 ± 0.02 ^bc^	0.26 ± 0.07 ^c^	0.27 ± 0.09 ^c^
Tyr	15.06 ± 0.18 ^a^	2.20 ± 0.11 ^b^	1.05 ± 0.05 ^c^	0.74 ± 0.03 ^d^	0.71 ± 0.07 ^d^
Phe	11.68 ± 0.42 ^a^	1.43 ± 0.13 ^b^	0.56 ± 0.01 ^c^	0.47 ± 0.03 ^c^	0.33 ± 0.04 ^c^
His	16.39 ± 0.94 ^a^	0.88 ± 0.05 ^b^	0.32 ± 0.04 ^c^	0.29 ± 0.02 ^c^	0.29 ± 0.03 ^c^
Lys	38.69 ± 1.66 ^a^	8.69 ± 1.41 ^b^	4.23 ± 0.72 ^c^	4.12 ± 0.38 ^c^	4.27 ± 0.13 ^c^
Arg	18.87 ± 0.29 ^a^	0.50 ± 0.06 ^b^	0.14 ± 0.03 ^c^	0.11 ± 0.02 ^c^	0.10 ± 0.01 ^c^
TAA	261.84 ± 1.36 ^a^	26.45 ± 2.05 ^b^	15.34 ± 0.52 ^c^	13.34 ± 0.29 ^c^	13.20 ± 0.25 ^c^
RAA (%)	100.00	10.11	5.86	5.10	5.05

Data represented as means ± SD (*n* = 3); a–d mean values within each row with different superscript letters were significantly different (*p* ≤ 0.05); db = dry basis; Asp = aspartic acid; Thr = threonine; Ser = serine; Glu = glutamic acid; Pro = proline; Gly = glycine; Ala = alanine; Cys = cysteine; Val = valine; Met = methionine; Ile = isoleucine; Leu = leucine; Tyr = tyrosine; Phe = phenylalanine; His = histidine; Lys = lysine; Arg = arginine; total amino acids indicate sum of 17 amino acids (TAA); remaining amino acids (RAA) = (total amino acids at t days × 100)/total amino acids at 0 days.

**Table 3:** Antioxidant activities, observed using the ABTS, DPPH, and FRAP methods, of SPIH-YCE samples during the humid–dry heating procedure at different incubation times.

Incubation Time (Days)	Method to Analyze Antioxidant Activity		
	ABTS (mg GAEs/100 g db)	DPPH (mg TEs/100 g db)	FRAP (mg FeSO_4_/100 g db)
0	596.19 ± 6.34 ^c^	140.87 ± 23.31 ^e^	724.04 ± 64.17 ^d^
5	736.41 ± 17.76 ^a^	676.42 ± 12.88 ^b^	4081.12 ± 57.16 ^b^
10	750.06 ± 13.82 ^a^	904.58 ± 54.91 ^a^	5102.92 ± 90.57 ^a^
15	674.63 ± 9.32 ^b^	582.38 ± 18.22 ^c^	4180.63 ± 124.80 ^b^
20	602.10 ± 13.21 ^c^	492.57 ± 54.65 ^d^	3834.32 ± 90.02 ^c^

Data represented as means ± SD (*n* = 3); a–e mean values within each column with different superscript letters were significantly different (*p* ≤ 0.05); db = dry basis; GAEs = gallic acid equivalents; TEs = Trolox equivalents; FeSO_4_ = ferrous sulfate.

## Data Availability

The original contributions presented in the study are included in the article. Further inquiries can be directed to the corresponding authors.
